# The Relationship between Leg Extension Angle at Late Stance and Knee Flexion Angle at Swing Phase during Gait in Community-Dwelling Older Adults

**DOI:** 10.3390/ijerph182211925

**Published:** 2021-11-13

**Authors:** Takasuke Miyazaki, Ryoji Kiyama, Yuki Nakai, Masayuki Kawada, Yasufumi Takeshita, Sota Araki, Hiroyuki Hayashi, Naoto Higashi, Hyuma Makizako

**Affiliations:** 1 Department of Physical Therapy, School of Health Sciences, Faculty of Medicine, Kagoshima University, Kagoshima 891-0175, Japan; k5588736@kadai.jp (T.M.); kawada@health.nop.kagoshima-u.ac.jp (M.K.); k1740552@kadai.jp (S.A.); makizako@health.nop.kagoshima-u.ac.jp (H.M.); 2 Department of Rehabilitation, Tarumizu Municipal Medical Center, Tarumizu Central Hospital, Kagoshima 891-2124, Japan; k2687318@kadai.jp; 3 Department of Mechanical Systems Engineering, Daiichi Institute of Technology, Kagoshima 899-4395, Japan; y-nakai@daiichi-koudai.ac.jp; 4 Graduate School of Health Sciences, Kagoshima University, Kagoshima 891-0175, Japan; 100hapiness@gmail.com (H.H.); monkey9.2018@gmail.com (N.H.)

**Keywords:** wearable sensor, joint angle, propulsion force, gait analysis

## Abstract

This study aimed to clarify the relationship between leg extension angle and knee flexion angle during gait in older adults. The subjects of this cross-sectional study were 588 community-dwelling older adults (74.6 ± 6.1 y). Segment angles and acceleration were measured using five inertial measurement units during comfortable gait, and bilateral knee and hip joint angles, and leg extension angle, reflecting whole lower limb extension at late stance, were calculated. Propulsion force was estimated using the increase in velocity calculated from anterior acceleration of the sacrum during late stance. Correlation analysis showed that leg extension angle was associated with knee flexion angle at swing phase and hip extension angle and increase in velocity at late stance (r = 0.444–508, *p* < 0.001). Multiple regression analysis showed that knee flexion angle at mid-swing was more affected by leg extension angle (β = 0.296, *p* < 0.001) than by gait speed (β = 0.219, *p* < 0.001) and maximum hip extension angle (β = −0.150, *p* < 0.001). These findings indicate that leg extension angle may be a meaningful parameter for improving gait function in older adults due to the association with knee kinematics during swing as well as propulsion force at late stance.

## 1. Introduction

A functional gait is integral to performing daily activities and enhancing the quality of daily life [1,2]. For safe and efficient walking, sufficient propulsion force at late stance, an appropriate knee flexion angle at swing phase, and increased toe clearance are required [3,4]. Propulsion force and knee flexion angle are decreased by aging and neurological and orthopedic conditions, resulting in low gait performance and an increased risk of falling [4,5,6,7,8]. Therefore, propulsion force and knee flexion angle are widely used as key parameters to assess gait quality in gait training and gait rehabilitation in older adults [4].

Leg extension angle, an angle consisting of a line connecting the hip joint with the ankle joint, and the laboratory’s vertical axis at late stance in the sagittal plane [9], is known to be related to propulsion force during gait [10]. Leg extension angle is also correlated with ankle moment and step length, and walking endurance [10,11,12]. Because leg extension angle is a visible parameter and includes kinetic and kinematic gait characteristics, it may a feasible and meaningful indicator for evaluating gait quality in clinical practice.

Knee flexion angle at swing phase is one of the major kinematic indicators that contributes to sufficient toe clearance [13]. Two-thirds of the knee flexion angle at swing phase during gait is achieved before toe off [14,15]. Knee flexion velocity at late stance is reported to contribute to knee flexion angle during swing phase [16,17]. Furthermore, lower limb muscle activity during the late stance phase contributes to peak knee flexion angle during swing phase [16]. The kinematics and kinetics of lower extremity at pre-swing affect knee flexion at swing phase. Therefore, understanding the dynamics throughout the walking cycle would be more important for gait training than focusing on a specific walking phase.

Approximately 30% of internal hip flexor moment at late stance is generated by the elastic energy of soft tissue, including the hip flexor muscle, joint capsule, and ligament [18]. Propulsion force during late stance would increase forward inertial force acting on the thigh segment, and it would contribute to knee kinetics during swing phase. Thus, an adequate leg extension angle, reflecting the propulsive force and lower limb kinematics at late stance, would affect knee flexion angle at swing phase during walking. However, the correlations among these remain unclear.

Therefore, the purpose of this study was to clarify the relationships between leg extension angle, propulsion force, and knee flexion angle during gait in community-dwelling older adults. We hypothesized that leg extension angle at late stance correlates with knee flexion angle at mid-swing as well as propulsion force at late stance. Leg extension angle may reflect the kinematic and kinetic gait quality during stance and swing phase. Our findings contribute to gait assessment and preventive interventions of community-dwelling older adults.

## 2. Materials and Methods

### 2.1. Participants

**Figure 1:**
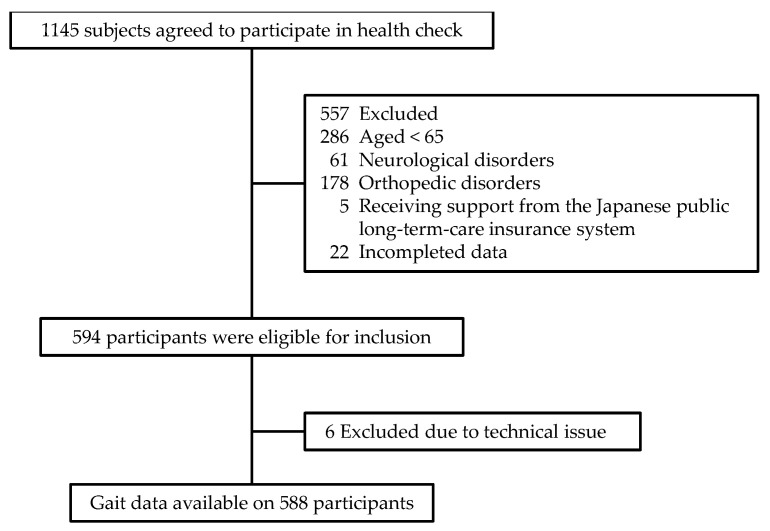
Subject flow diagram from initial contact through to study completion.

The present cross-sectional study used data from the Tarumizu Study 2018, which was conducted in cooperation with Kagoshima University (Faculty of Medicine), Tarumizu City Office, and Tarumizu Chuo Hospital; it was held from June to December 2018 as a community-based health check survey. Individuals selected to participate in the Tarumizu Study 2018 were chosen from among the older adults living in Tarumizu City, a city close to Kagoshima, Japan. Subject enrollment involved sending e-mail messages to all citizens aged 40 years and older; 1145 subjects agreed to participate in the Tarumizu Study 2018 and underwent a health check. The inclusion criteria of this study were as follows: living in Tarumizu City, aged ≥ 65 y (years), and able to walk without walking aids. We excluded participants with neurological and orthopedic disorders, such as stroke, Parkinson’s disease, dementia, depression, lower limb fractures, and osteoarthritis, those receiving support from the Japanese public long-term-care insurance system using self-report questionnaires, and those with missing data. As a result of recruitment, 588 community-dwelling older adults (363 female, 74.6 ± 6.1 y) participated in this study (Figure 1). Informed consent was obtained from all participants before their inclusion in the study, and the ethics committee of the Faculty of Medicine, Kagoshima University approved the study protocol (ref no. 170103).

### 2.2. Gait Measurement

Participants walked at a comfortable velocity along a 14 m straight walkway twice. Bilateral hip and knee joint angles were measured during gait using five inertial measurement units (IMUs; Mtw Awinda, Xsens, Enschede, Netherlands) with sampling rates of 100 Hz. IMUs consisted of a 3D rate gyroscope, a 3D accelerometer, and a 3D magnetometer, and could calculate the acceleration and Euler angle in the global coordinate system using MT manager software (4.7.2, Xsens, Enschede, Netherlands). This software used the Kalman filter to estimate these measurements from magnetic and inertial data. The reliability of IMU has been reported previously [19]. IMUs were fixed above the posterior sacrum and anterior to bilateral thighs and shanks by elastic belts. IMUs were attached frontally and vertically against the frontal plane, where possible, and were calibrated so that the vertical direction of the IMU coordinate system was in line with gravity during static standing by MT manager [20]. We also measured the length of the right thigh and shank with a tape measure and the gait speed in the middle 10 m of the 14 m straight walkway with a stopwatch.

### 2.3. Data Analysis

**Figure 2:**
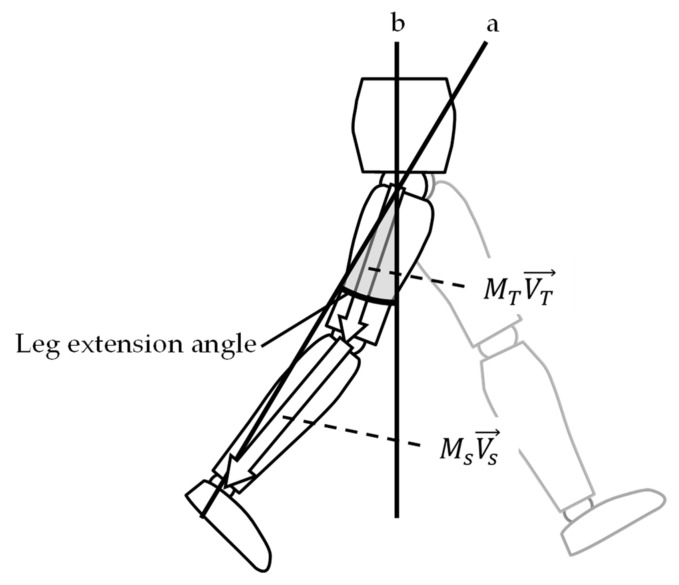
Estimation of the leg extension angle. Leg extension angle was calculated as an angle consisting of a line connecting the hip joint with the ankle joint (**a**), and the laboratory’s vertical axis (**b**) at late stance in the sagittal plane. $M_{T}$, tilt angle matrix of thigh segment; $M_{s}$, tilt angle matrix of the shank segment; $\vec{V_{T}}$, vector of the thigh segment; $\vec{V_{s}}$, vector of the shank segment.

A third-order Butterworth low-pass filter was performed on the data measured by IMU with a 20 Hz cutoff frequency to reduce the noise. Hip and knee joint angles were calculated as relative Euler angles among the pelvis, thigh, and shank segments, as measured by IMU. The location of the knee joint and the ankle joint relative to the hip joint was estimated from the tilt angle matrix measured by IMU and the vector of the thigh and shank segment coordinated by the segment length (Figure 2). Leg extension angle was then calculated from the location of the ankle joint relative to the hip joint in the sagittal plane [20]. Joint angle and leg extension angle were adjusted to measure zero at quiet standing. Previous studies have confirmed the validity of using IMUs to determine these gait parameters [20,21].

Propulsion force is usually assessed via anterior ground reaction force at late stance. We preliminarily confirmed that the impulse of anterior ground reaction force is closely correlated with the increase in velocity calculated from the integration of the anterior acceleration measured in IMU fixed on the sacrum [20]. Thus, the increase in velocity at late stance was calculated as an indicator of propulsion force.

**Figure 3:**
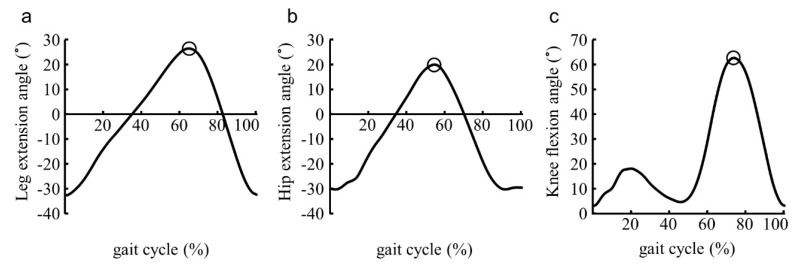
Representative waveform of the lower limb joint angle during gait. (**a**) Leg extension angle and (**b**) hip extension angle during late stance, and (**c**) knee flexion angle at swing phase were calculated from the tilt angle measured by inertial measurement units. Blank circles indicate the maximum value of each angle.

Maximum hip extension angle and leg extension angle during late stance, and knee flexion angle at swing phase were calculated during the five walking cycles of bilateral lower extremities in the middle of the 14 m distance (Figure 3). Data processing was performed using MATLAB R2020a (Mathworks Inc, Natick, MA, USA) mathematical software.

### 2.4. Statistical Analysis

The mean of variables of bilateral lower extremities during 10 strides was taken as the representative value. Pearson’s correlation analysis was conducted to examine the relationships between leg extension angle and other gait parameters. Previous studies have reported that age, sex, and gait speed alter the kinematics and kinetics of lower limbs during gait [4,22,23,24]. Thus, simultaneous multiple regression analysis was performed to determine the relationships among leg extension angle, knee flexion angle at mid-swing phase, and hip extension angle at late stance after controlling simultaneously for age, sex, and gait velocity. All statistical analyses were performed using SPSS 25 (IBM, New York, NY, USA), and the significance level was set at 5%.

## 3. Results

Gait speed was 1.28 ± 0.20 m/s; leg extension angle was 23.5 ± 3.5°; knee-flexion angle at mid-swing was 63.4 ± 7.8°; hip extension angle at late stance was 12.4 ± 4.5°; and increase in velocity at late stance was 0.38 ± 0.09 m/s.

**Figure 4:**
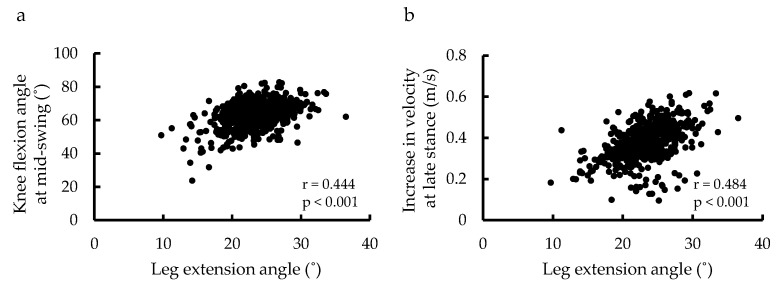
Scatter plot of leg extension angle, knee joint angle, and increase in velocity. Relationships between leg extension angle and knee flexion angle at mid-swing (**a**), and between leg extension angle and increase in velocity at late stance (**b**).

Correlation analysis showed that leg extension angle was associated with knee flexion angle at swing phase (r = 0.444, *p* < 0.001) and hip extension angle (r = 0.508, *p* < 0.001) and increase in velocity (r = 0.484, *p* < 0.001) at late stance (Table 1; Figure 4). Knee flexion angle at mid-swing was significantly correlated with all variables at late stance, especially with leg extension angle (Table 1).

Multiple regression analysis adjusted for age, sex, and gait speed showed that knee flexion angle at mid-swing was correlated with leg extension angle (β= 0.296, *p* < 0.001), hip extension angle at late stance (β = −0.150, *p* < 0.001), and gait speed (β = 0.219, *p* < 0.001) (R^2^ = 0.323; Table 2).

## 4. Discussion

This study examined the relationships among leg extension angle, increase in velocity at late stance, and knee flexion angle at swing phase in community-dwelling older adults. Leg extension angle at late stance was correlated with knee flexion angle at mid-swing and the increase in velocity at the late stance. These findings indicate that leg extension angle reflects the gait quality during stance and swing phase, supporting our hypothesis. Leg extension angle is a meaningful indicator of gait quality in older adults.

Gait velocity, hip extension angle at late stance, and knee flexion angle at mid-swing in the present study were 1.28 m/s, 12.4°, and 63.4°, respectively. These variables were similar to those in previous studies, which reported 1.3 m/s for gait velocity [25], 14° for hip extension angle at late stance [26], and 65° for knee flexion angle at mid-swing [6,27].

Correlation analysis indicated positive relationships among leg extension angle, hip extension angle, increase in velocity at late stance, and knee flexion angle at mid-swing. This result is consistent with previous studies investigating the relation between leg extension angle and propulsion force [10,28]. Propulsion force during late stance likely increases forward inertial force acting on the thigh segment and contributes to knee kinetics during swing phase.

Multiple regression analysis showed that knee flexion angle at mid-swing was most strongly correlated with leg extension angle at late stance, even after excluding the effects of age, sex, and gait velocity. Leg extension angle may contribute to an increase in propulsion force and elastic energy of hip flexors, capsules, and ligaments, relating to increased inertial forward force in the thigh. A previous study reported that approximately 30% of internal hip flexor moment at late stance is generated by the elastic energy of the soft tissue, including the hip flexor muscle, joint capsule, and ligament [18]. Multiple regression analysis also showed the negative contribution of hip extension angle to knee flexion angle at mid-swing. This result was contrary to the result of the correlation analysis. Considering that the hip begins to flex with knee flexion at pre-swing, too-large hip extension would delay the initiation of knee flexion. Thus, leg extension angle, rather than hip extension angle, reflects the whole lower limb kinematics at late stance and is important for sufficient knee flexion at mid-swing as well as for safe walking.

Leg extension angle at late stance is related to propulsion force during walking [10,28]. Furthermore, this study showed that leg extension angle was correlated with knee flexion angle at mid-swing. The previous report showed that fallers had a more reduced knee flexion angle during the swing phase than that of non-fallers [28]. Thus, improvement in leg extension angle could alter the gait kinematics and kinetics throughout the walking cycle, allowing safe and efficient walking. Gait assessment using wearable sensors is important in clinical practice [29], and leg extension angle is a useful parameter, because older adults can easily recognize and improve it by themselves during daily walking [30,31,32]. Therefore, therapists should focus on these parameters during gait assessment and instruction using IMU.

This study had some limitations. First, although ankle function affects leg extension angle [10], we did not measure ankle movement due to concerns over estimation of the location of the metatarsal joint by IMU. Further study is needed to examine these relationships, including ankle function. Second, propulsion force was estimated by the increase in velocity, not by the anterior ground reaction force. Further study, including kinetic analysis, is needed to examine the usefulness of the leg extension angle to assess gait function in older adults.

## 5. Conclusions

In this study, we examined the relationships among leg extension angle and propulsion force at late stance, and knee flexion angle at mid-swing. This study showed that leg extension angle at late stance correlates with knee flexion angle at mid-swing as well as an increase in velocity at late stance. The present study suggests that leg extension angle may be a meaningful gait parameter that leads to efficient and safe walking in older adults. Therefore, therapists should first assess and then facilitate leg extension angle at late stance during gait training for older adults.

## Figures and Tables

**Table 1:** Correlation coefficients between leg extension angle and gait parameters at late stance and swing phase.

Gait Parameter	Late Stance			Mid-Swing
	Leg Extension Angle	Hip Extension	Increase in Velocity	Knee Flexion
**Late stance**				
Leg extension angle	-	0.508 **	0.484 **	0.444 **
Hip extension		-	0.300 **	0.126 **
Increase in velocity			-	0.344 **
**Mid-swing**				
Knee flexion				-

** *p* < 0.01.

**Table 2:** Multiple regression analysis of knee flexion angle at mid-swing and gait parameters at late stance.

Variables	Unstandardized Regression Coefficient (B)	Standardized Regression Coefficient (β)	*p*
Leg extension angle	0.663	0.296	<0.001
Hip extension angle	−0.264	−0.150	<0.001
Gait speed	7.623	0.219	<0.001
Age	−0.342	−0.265	<0.001
Sex	−1.107	−0.060	0.047

## Data Availability

The data used to support the findings of current study are available from the corresponding author upon request.
